# Iliac vein stenting in a patient with lower extremity swelling resulting from diffuse pelvic mass: A case report

**DOI:** 10.34172/jcvtr.2020.57

**Published:** 2020-12-23

**Authors:** Niki Tadayon, Sina Zarrintan, Seyed Masoud Hosseini, Seyed Mohammad Reza Kalantar-Motamedi

**Affiliations:** ^1^Division of Vascular & Endovascular Surgery, Department of General & Vascular Surgery, Shohada-Tajrish Medical Center, Shahid Beheshti University of Medical Sciences, Tehran, Iran; ^2^Phlebology Research Group, Shahid Beheshti University of Medical Sciences, Tehran, Iran; ^3^Cardiovascular Research Center, Tabriz University of Medical Sciences, Tabriz, Iran

**Keywords:** Iliac Vein, Venous Stent, Pelvic Mass, Venoplasty

## Abstract

We report a 66-year-old male patient with severe right lower extremity swelling resulting from diffuse pelvic mass with compression on right external iliac vein. The patient had papillary urothelial carcinoma of bladder seven years ago and radical cystectomy and ureterostomy was performed. Recurrence of malignancy had occurred five years after the operation. The patient had also bilateral diffuse lung metastasis. The external iliac vein had severe stenosis and invasion of pelvic mass into the vein was evident on venography. Venoplasty of external iliac vein was performed throughout the stenosis. A venous stent of 80 mm length and 12 mm diameter was introduced over the guidewire and deployed in the external iliac vein. Dramatic clinical response was evident since postoperative day two. Swelling of right lower extremity was resolved dramatically on three-month and six-month follow-up visits. We believe that endovascular venous recanalization of iliac veins is feasible and safe in patients with unresectable and diffuse pelvic masses.

## Introduction

Chronic venous involvement and occlusion is relatively common in cancer patients.^1^ Malignant pelvic masses may compress iliac veins. This may result in venous occlusion and lower extremity swelling.^2^ Treatment of venous compression is necessary to alleviate signs and symptoms especially in patients with unresectable pelvic tumors.^3^ Endovascular venoplasty and venous stenting is a useful technique to restore the venous flow and palliate the symptomatic patients with pelvic mass.^4-6^

O’Sullivan et al^7^ reported a series of nine patients with venous obstructions of lower extremity resulting from extrinsic compression from a malignant or other masses in the pelvis. They used iliofemoral venous stenting to re-establish the venous flow. They reported a clinical patency rate of 85%. In another report from O’Sullivan et al^8^, 62 patients with metastatic pelvic disease and lower extremity swelling were managed by venous stenting. They reported considerable alleviation of symptoms and satisfaction of studied patients. Similar favorable outcomes have been reported by Maleux et al^3^ and Nazarian et al.^9,10^

Herein, we report a case of lower extremity swelling resulting from diffuse pelvic mass. Successful iliac venous stenting and reestablishment of venous flow was performed.

## Case Presentation

A 66-year-old male patient with history of previous papillary urothelial carcinoma of bladder was referred to our vascular surgery clinic. The patient had undergone radical cystectomy and ureterostomy seven years ago. Recurrence of malignancy had occurred five years after the operation. The patient had a mass at right side of pelvis compressing the right external iliac vein on magnetic resonance imaging. The patient had also bilateral diffuse lung metastasis on lung computed tomography. The patient had received 12 courses of chemotherapy. Resection of pelvic mass was not indicated because of diffuse lung metastasis. Right lower extremity had severe swelling. Above knee and thigh of the affected limb had larger diameter of 6.5 and 9.5 cm than the contralateral limb respectively. The patient suffered from right lower extremity pain and had a venous ulcer at right medial malleolus.

**Figure 1:**
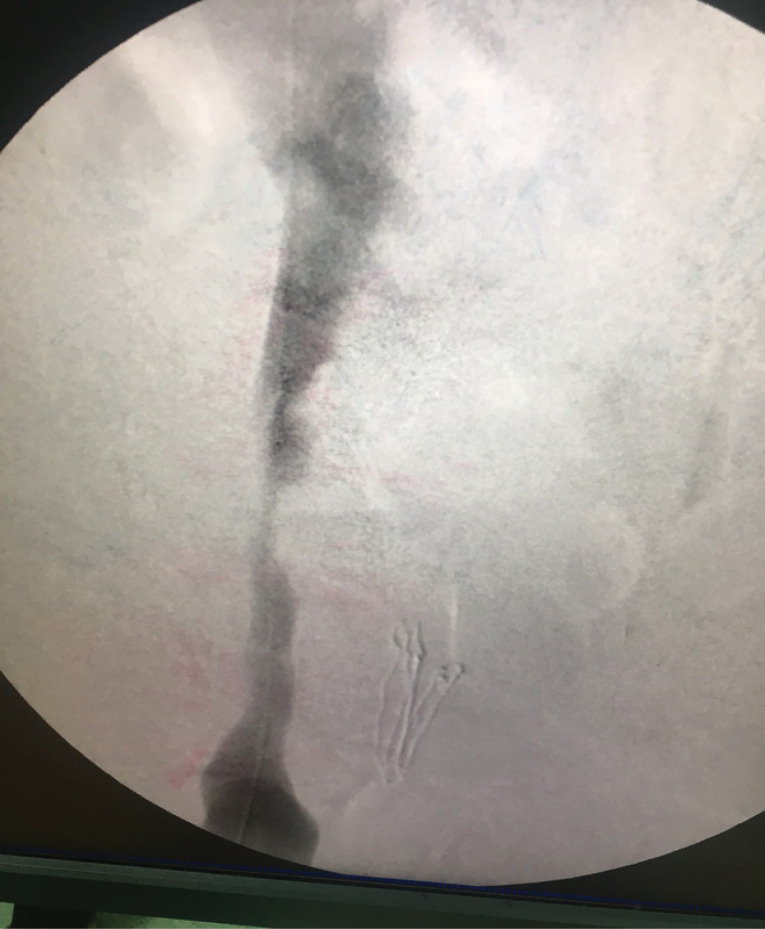
Severe stenosis of right external iliac vein secondary to compression from diffuse right pelvic mass

**Figure 2:**
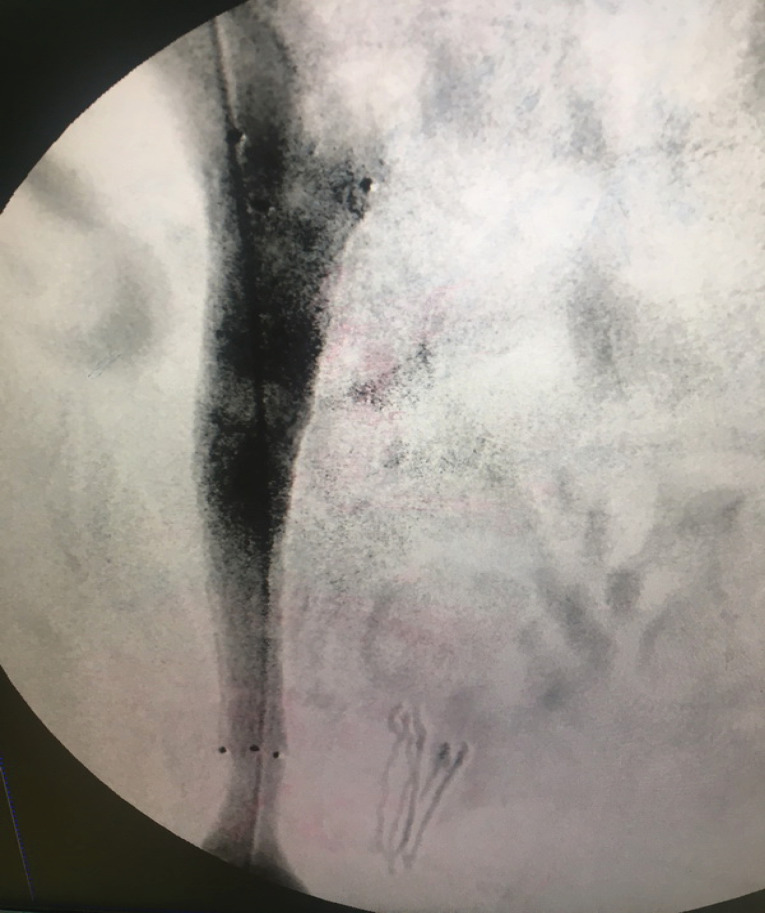
Venoplasty and stenting of right external iliac vein

We did venography to reveal the underlying cause of venous stasis in right lower extremity. We did the imaging on prone position. A 5F sheath was introduced through the right popliteal vein. The external iliac vein had severe stenosis and invasion of pelvic mass into the vein was evident. This procedure was done under close monitoring of vital signs and local anesthesia. We transformed the patient to supine position to cannulate right superficial femoral vein (SFV). An 8F sheath was introduced into SFV by ultrasound guidance and Seldinger technique 10cm below the inguinal ligament. The reason for changing the position of the patient was that the procedure of stenting is painful. We wanted to do the procedure under deep sedation and it was not possible to do this in prone position because of general condition of the patient. A repeat venogram was obtained at supine position and similar results were found (Figure 1). Venography was repeated at 45 and 90 degrees of left lateral oblique views and severe stenosis and tumor invasion were confirmed. A 0.035 hydrophilic standard guidewire was introduced through the sheath and the stenosis was crossed. Then a 40 mm length and 10 mm diameter CONQUEST balloon (BARD) was introduced over the guidewire. Venoplasty of external iliac vein was conducted throughout the stenosis and at proximal and distal parts. Then a venous stent of 80 mm length and 12 mm diameter (VENOVO, BARD) was introduced over the guidewire and deployed in the external iliac vein with the stenosis trapped inside the length of the stent. The completion venogram illustrated dilatation of the stenosis and reestablishment of flow of contrast material through the external iliac vein (Figure 2). Then a 40 mm length and 12 mm diameter CONQUEST balloon (BARD) was introduced over the guidewire. Balloon dilatation was done through the stent and its proximal and distal landing zones. 7500 units of intravenous heparin was administered during the procedure. A loading dose of 300mg Clopidogrel was administered to the patient after the procedure at the recovery theater. The patient was anticoagulated by heparin for 48 hours and Clopidogrel 75mg daily was also administered during that period. Rivaroxaban 10mg per 12 hours and Clopidogrel 75 mg daily was administered on discharge. The patient was on Aspirin 80 mg daily before the procedure and we advised him to continue the same dose.

Dramatic clinical response was evident since postoperative day two. The patient was followed after three and six months. Swelling of right lower extremity was resolved dramatically and the venous ulcer at right medial malleolus was healed. A venous duplex study was done at follow-up visits. Normal venous flow was present.

## Discussion

We report a case of iliac venous stenting in a patient with right external iliac vein compression resulting from diffuse pelvis mass. The mass was recurrence of papillary urinary carcinoma of bladder. Severe lower limb swelling and venous ulcer responded dramatically to reestablishment of iliac venous flow via stenting. We believe that endovascular venous recanalization of iliac veins is feasible and safe in patients with unresectable and diffuse pelvic masses. Although recurrence of stenosis is possible, quality of life and symptom relief is of potential clinical value in affected patients.

In a recent report, Taslakian et al presented a case of right lower extremity edema resulting from a retroperitoneal mass. The mass was recurrence of a previous bladder and prostate cancer. The compression of iliac veins caused deep vein thrombosis, venous obstruction and limb swelling. They used venous stenting and the symptoms disappeared.^1^ We managed our case with a similar procedure and successful results were obtained. Recanalization of the vein is possible through the mass. Although recurrence is possible, palliation of the symptoms in an end-stage cancer patient is invaluable.

Hama et al reported a case of 70-year-old woman with ureteral carcinoma with occlusion of bilateral iliac veins and inferior vena cava (IVC). They used successful stenting and reestablishment of venous flow.^2^ The reported case of Hama et al was a bilateral venous stenosis. However, our patient and the reported case of Taslakian et al^1^ were unilateral venous stenosis. We believe that venous stenting for reestablishment of venous flow in malignant venous compression can be approached by stenting even in bilateral involvements. IVC stenting is also feasible. Maleux et al also reported similar cases of iliac or IVC cancer-related iliocaval obstructive diseases which were successfully managed by stenting.^3^

Pelvic lymphadenopathy resulting from malignancies may also cause iliac venous compression and lower limb swelling. Takai et al^6^ reported a case of right external iliac vein compression resulting from lymph node involvement in a female patient with endometrial cancer. The patient was managed with venous stenting. Dramatic improvement of quality of life was reported. Our case had also involvement of right external iliac vein. Depending on the compressing mass, distal iliofemoral venous involvement^6-8^ or proximal iliocaval involvement^2^ may be present. In both locations, venous stenting and recanalization is safe and feasible. However, proximal extension and IVC involvement may make the procedure more difficult.

## Conclusion

In conclusion, alleviation of symptoms in lower extremity swelling in cases of pelvic malignant compression of iliac veins are safe and feasible. Flow of common and external iliac veins and more distal iliofemoral veins can be reestablished by venoplasty and stenting. Although technically difficult, IVC stenting is also possible. Although venous stenting is a challenging and controversial issue in chronic venous insufficiency, it is of valuable clinical interest in end-stage patients with unresectable pelvic mass and subsequent iliac venous stenosis and occlusion. This procedure is a palliative treatment to restore venous blood flow and provide clinical response.

## Acknowledgements

The authors would like to acknowledge Dr. Annis Shahnaee for her help during the preparation of this manuscript.

## Competing interest

None.

## Ethical approval

Informed consent has been obtained from the patient to report the case.

## Funding

None.
